# Diseases of House Painters

**Published:** 1869-10

**Authors:** 


### DISEASES OF HOUSE PAINTERS.

Painters, more than any other class of artizans, suffer from disease. This is owing to the fact that their hands, arms, faces and clothing are constantly daubed with the paint with which they are employed, and is absorbed through the skin, giving rise to paralysis, colic, and kidney disease.

Lead, when taken into the system, is poisonous. The painter, when contaminated with it becomes dizzy, debilitated, and "low spirited." He loses all energy and ambition, suffers from occasional attacks of water-brash and colic, until he finally becomes prostrated from paralysis of one limb, one side of the face, one-half of his person or throughout his entire body.

Some are able to follow the business for several years without contamination, but sooner or later the symptoms of poisoning will overtake and prostrate them.

Many hasten the disease by rushing to their meals without washing the paint from their hands, and in this manner mixing paint with their food; while others endanger their lives by wearing clothing completely saturated with repeated daubings of paint.

Painters could render their business less perilous by having their clothing made of fabrics that could be easily washed, being careful to have them thoroughly renovated every week; by washing every particle of paint from their persons before going to meals; by bathing their hands every night in a strong decoction of oak bark; by keeping the hair cut short and wearing thin and clean caps; by removing the characteristic deposit upon the teeth with weak oak-bark-decoction, applied daily with a brush; and by eating plentifully of fatty substances and drinking new milk in large quantities.

Notwithstanding repeated warnings of the approach of symptoms of lead poisoning, men will persist in their work without taking the necessary precautions to avert a fatal termination. The moment any of the symptoms described manifest themselves, work should be suspended for a season, and a thorough renovation of person and clothing had. We have recently seen several severe cases of poisoning from neglecting such advice, and were called upon to attend one case that terminated fatally on the first day after the attack, which has led us to pen these lines, as a warning to a large class of workmen, constantly employed in the manufacture of lead and paint, and to those known as painters.

